# Kimura's disease with bilateral parotid involvement: a common presentation with an uncommon diagnosis

**DOI:** 10.1016/j.bjorl.2021.03.011

**Published:** 2021-04-20

**Authors:** Hui Yan Ong, Mimi Ezreena Esa, Jia Ji Ng, Shahawiah Abdul Wahab, Santhi Kalimuthu

**Affiliations:** a Tengku Ampuan Rahimah Hospital, Ministry of Health, Department of Otorhinolaryngology, Klang, Selangor, Malaysia; b University Malaya Medical Centre, Kuala Lumpur, Malaysia

## Introduction

Kimura's disease is an uncommon chronic inflammatory condition with unknown etiology. Patients with Kimura's disease usually present with solitary or multiple soft tissues masses in the head, neck, and limbs, which often recur after treatment.^1^ It can be misdiagnosed and there is presently no established standard treatment.^1^ We present a case of a middle-aged man who has had recurrent parotid swellings, being eventually diagnosed as Kimura's disease via excisional biopsy of the cervical lymph nodes.

## Case report

We present a case of a 43 year-old man with no known medical illness, who presented with bilateral parotid swelling for the past 5 months. He started noticing painless swelling over the right parotid region. He did not seek treatment until the left parotid region also developed swelling, gradually increasing in size. There was no febrile episode, toothache, nor ear or nasal symptoms. There were also no obstructive symptoms. He was tolerating oral intake well, with no constitutional symptoms. He was given multiple courses of oral antibiotics to no avail. He recalled a similar swelling over the right neck a few years ago, when he was treated in a private medical center. The swelling was removed surgically, and he was told that it was a benign growth. He was discharged and there were no subsequent followups. He was eventually referred to our clinic by the primary care center for further management. Full pulmonary tuberculosis workup has also been done for him in primary care setting to rule out this rather common infection in our country.

Upon review in our clinic, he was alert, pink with good nutritional status. There was no facial asymmetry. On the right, there was a parotid swelling measuring 15 cm by 10 cm, which was firm, non-tender and slightly mobile, non-adherent to the skin. There was a healed postauricular scar, which was believed to be the operative site of previous swelling. On the left, there was a parotid swelling measuring 5 cm by 6 cm, which was also firm, non-tender and slightly mobile. There was also an enlarged, non-tender, mobile lymph node measuring 2 cm by 3 cm. Oropharyngeal and otoscopic examinations were also normal. Flexible nasopharyngolaryngeal examination revealed clear bilateral fossa of Rossenmullers and normal laryngeal structures.

**Figure 1:**
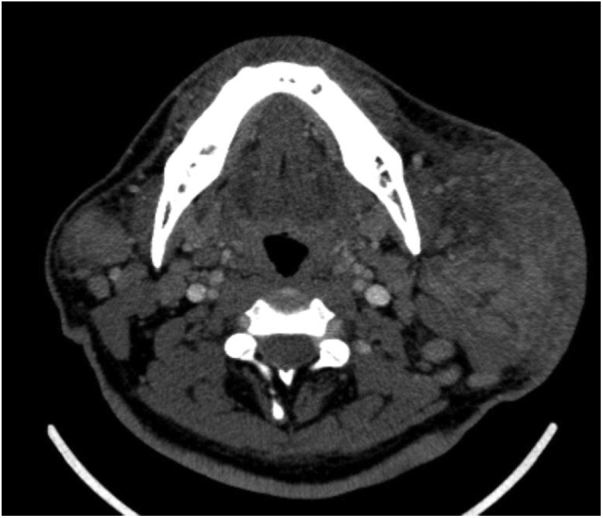
Computed tomography of the neck (axial view) showing extensive fat streakiness of overlying subcutaneous tissue plane with skin involvement over the right. There is no focal enhancing collection seen within the glands.

**Figure 2:**
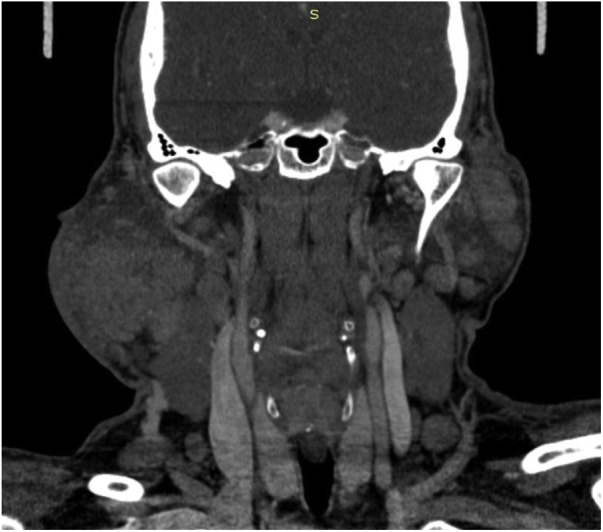
Computed tomography of the neck (coronal view) showing bilateral diffuse enlargement of the parotid glands, right more than left, multiple nodules of varying sizes in the superficial and deep lobe of both glands. Both the internal jugular veins and carotid vessels are preserved.

Blood investigations revealed elevated white blood cell counts of 20.43 × 10^9^/L with eosinophil predominance, which was as high as 62.0%. His renal profile was normal. Computed tomography imaging of the neck revealed diffuse heterogenous enlargement of bilateral parotid glands, with multiple nodules of varying sizes in both superficial and deep lobes of the parotid glands. There was extensive fat streakiness of overlying subcutaneous plane with skin involvement, particularly on the right (Fig. 1). The plane between the right masseter and sternocleidomastoid muscles was also not well delineated. There was no focal enhancing collection within the parotid glands (Fig. 1). There was multiple enhancing enlarged nodes seen at level I, II, III, IV and V, bilateral supraclavicular region and bilateral paratracheal region (Fig. 2). In view of the bilateral involvement of the parotid glands, patient's age, sex and history of painless swellings, the imaging features could be suggestive of Kimura's disease. Nodal metastases and lymphoma were unable to be excluded.

Fine needle aspiration for cytology was done on both the right and left parotid glands. The smears showed polymorphous population of lymphoid cells with predominance of eosinophils. The differential diagnosis given included Kimura lymphadenopathy, Hodgkin Lymphoma, drug reaction as well as parasitic infection. In order to exclude neoplastic process and for proper histological assessment, excision of the right cervical lymph node was done. The right level III cervical lymph node was removed. He recovered well postoperatively and the wound healed well. The histopathological analysis of the lymph node eventually confirmed the diagnosis of Kimura's disease (Fig. 1). He was referred to the dermatology department for further management. With steroid therapy, the bilateral parotid swellings gradually reduced in size and the appearance was acceptable for the patient. He was not keen on further surgical intervention.

## Discussion

Kimura's disease is an uncommon chronic inflammatory disorder with less than 300 cases reported to date.^1^ It was first reported in China in 1937, by Kim and Szeto under the term ‘eosinophilic hyperplastic lymphogranuloma’.^2^ It is currently known as Kimura's disease after being systematically described as ‘unusual granulations combined with hyperplastic changes in lymphoid tissue’ by Kimura et al. in 1948.^3^ Most cases reported occurred in East Asians and Southeast Asian populations. The etiology is unknown, though many consider it as an IgE-mediated type 1 inflammatory disease, evidenced by the increased eosinophil count and IgE levels in the peripheral blood.^1^ Inflammation is the predominant feature of this disease.^4^ To date, there is no correlation found between Kimura's disease with infective agents such as tuberculosis, fungal, bacteria or viral infections.^1^, ^4^

Kimura's disease is mostly seen in young Asian men, with a male: female ratio of 3:1, aged between 27 and 40 years old,^5^ though the minimum age reported was as young as 5 years old.^1^ Kimura's disease usually affects the skin, soft tissues and lymphatics of the head and neck region, namely the parotid and submaxillary glands.^6^ Limbs, trunks, retroperitoneum and groin are rarely affected.^6^ However, for non-Asian patients, involvement of the salivary glands is uncommon.^1^ Bilateral involvement of the parotid glands is uncommon^7^: Warthin's tumors, being the most common bilateral parotid tumors, should be considered first. Our patient initially presented with right neck swelling a few years before, for which surgical excision was done. He presented to us this time with bilateral parotid gland involvement. Warthin's tumors were considered in view of his age and smoking history, as well as the prevalence of these tumors. However, blood eosinophilia and the subsequent cytology results were suggestive of Kimura's disease instead.

Kimura's disease usually manifests as solitary or multiple firm, painless, subcutaneous swellings, which increase in size progressively. The swelling size varies, ranging from 1 to 7 cm.^1^ The skin is usually not inflamed.^8^ Occasionally, there may be skin itching, skin pigmentation, thickening, local erosion or even ulceration.^1^ Regional lymphadenopathies are usually present.^1^ Renal involvement is also found in 60% of patients with Kimura's disease. Patients may develop proteinuria, nephrotic syndrome, and other renal abnormalities such as membranous nephropathy and mesangioproliferative glomerulonephritis. Renal disorders may occur prior to, simultaneously or following this disease. Our patient reported neck swellings for years and was not properly followed up. Despite that, he had no renal involvement.

The diagnosis of Kimura's disease is challenging as there are no characteristic features which are specific for the disease, both radiographically and cytologically.^4^ Iwai et al. concluded that in Asian patients with parotid swelling and eosinophilia of more than 10.5% are diagnostic of Kimura's disease.^9^ Biochemical profiles of patients including marked eosinophilia and raised serum Ig E levels should raise suspicion of this disease.^1^ Imaging studies such as computed tomography or magnetic resonance imaging may reveal a wide range of lesions with lymphadenopathy which are not conclusive.^1^ Fine needle aspiration for cytology can be done, but incisional or excisional biopsy will be able to provide ample tissue for a more accurate histopathological diagnosis.

**Figure 3:**
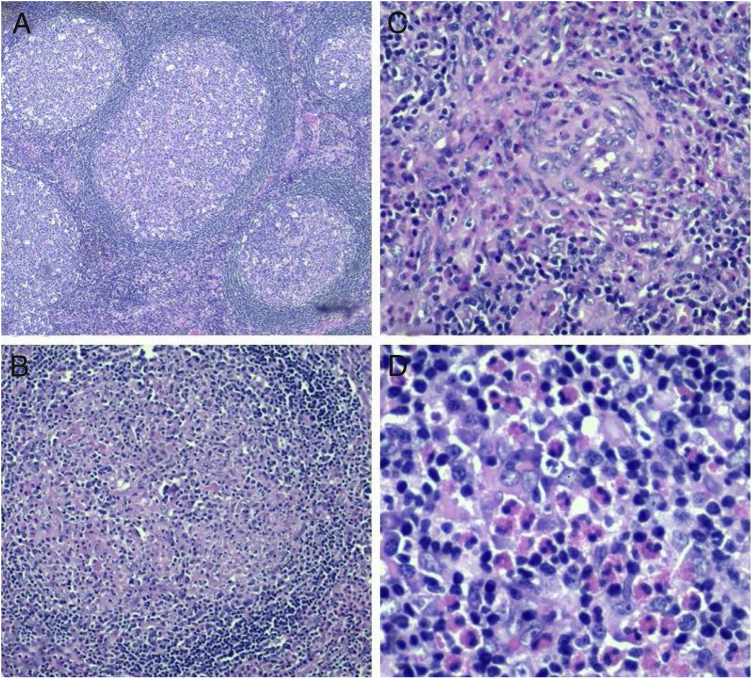
Histological slides of right cervical lymph node. (A) Reactive lymphoid follicles with prominent germinal centers (4×); (B) proteinaceous material within germinal centers with surrounding eosinophils (10×); (C) proteinaceous material within germinal center (20×); (D) interfollicular eosinophils (40×).

The histopathological characteristic features of Kimura's disease are the destruction of the normal architecture and replacement with heavy lymphoid infiltrate, together with lymphoid follicles with prominent germinal centers.^6^ The interfollicular infiltrates are rich in eosinophils, lymphocytes, plasma cells and mast cells.^5^ Vascular proliferation and fibrosis may also be observed sometimes.^10^ In this case, the biopsy of the enlarged lymph nodes revealed a thick capsule with preserved architecture (Fig. 3). There are scattered enlarged lymphoid follicles with prominent germinal centers. Fibrosis and proteinaceous materials are noted in the germinal centers. Interfollicular areas also showed dilated sinusoids with venules exhibiting plump endothelial cells. There were eosinophilia and micro-abscesses seen occasionally. There was no cellular atypia and evidence of malignancy.

Angiolymphoid hyperplasia with eosinophilia is one of the important differential diagnosis of Kimura's disease. This disease manifests similarly with plaques or skin nodules around the head and neck region, but they are more common in middle-aged women.^6^ Blood eosinophilia and elevated serum IgE levels are rare in angiolymphoid hyperplasia, but histopathologically, eosinophils infiltrates and vascular proliferation are also present.^10^ On the other hand, Kikuchi-Fujimoto disease may also be considered, but it differs histologically, whereas there is necrosis with large numbers of different histiocytes surrounding the necrosis areas.^10^ Other more common diseases in which patients present with multiple neck swellings and lymphadenopathies should always be considered, including lymphomas, tuberculosis, and even malignancies, especially in patients of older age group. Autoimmune diseases such as Sjogren's disease, granulomatosis with polyangiitis and sarcoidosis are uncommon in our local setting, but should be considered as well.

There is presently no standard guideline for treatment of this disease.^1^ The main aims of treatment are to maintain functionality with acceptable cosmesis, while preventing recurrences and long-term complications such as nephritis.^1^ Surgical excision is the most favorable method as it requires less time compared to other treatments and allows accurate histopathological assessment.^7^ However, there may be recurrence and risks of facial nerve injury during surgery. In our case, despite surgical excision, patient experienced recurrence of neck swellings. Based on the investigational results which were more suggestive of Kimura's disease, we decided to perform excisional biopsy of the cervical lymph nodes for confirmation of the diagnosis, instead of excision of the parotid masses or parotidectomy. This is because patient was asymptomatic and preferred a more conservative approach. In a similar case reported by Woo SH et al., where the patient also presented with bilateral parotids involvement, surgical excision with partial parotidectomy was done, as patient was complaining of pain and wished to have immediate treatment effect.^7^

Radiotherapy and immunosuppressive therapy such as corticosteroids, cyclosporins, or other cytotoxic agents have also been used.^1^ These therapies may be able to reduce the size of the masses, but usually do not remove them completely, and relapses are common after discontinuation of therapy.^7^ Nonetheless, oral corticosteroids are recommended for patients with nephrotic syndrome, and postoperative radiation therapy may also be helpful in preventing relapses.^1^ Anti-Ig E therapy has also been introduced and the effect was reported to be favorable. The overall prognosis of Kimura's disease is still considered good though recurrence rate is up to 25%,^1^ as it is benign and self-limiting, with no malignant transformation reported as of now. Despite this, patients should be followed up to monitor recurrences as well as possible systemic complications.

## Conclusion

Kimura's disease is uncommon, and the diagnosis can only be accurately confirmed by histopathological assessment. Patients should be well informed regarding the benign and recurrence nature of this disease. Treatment should be individualized as there are advantages and disadvantages of each modality.

## Conflicts of interest

The authors declare no conflicts of interest.
